# Tissue Culture and Somatic Embryogenesis in Warm-Season Grasses—Current Status and Its Applications: A Review

**DOI:** 10.3390/plants11091263

**Published:** 2022-05-07

**Authors:** Melody Ballitoc Muguerza, Takahiro Gondo, Genki Ishigaki, Yasuyo Shimamoto, Nafiatul Umami, Pattama Nitthaisong, Mohammad Mijanur Rahman, Ryo Akashi

**Affiliations:** 1 Faculty of Agriculture, University of Miyazaki, 1-1 Gakuenkibanadai-Nishi, Miyazaki 889-2192, Japan; melody.muguerza@gmail.com (M.B.M.); gishigaki@cc.miyazaki-u.ac.jp (G.I.); hi_tei05428@yahoo.co.jp (Y.S.); rakashi@cc.miyazaki-u.ac.jp (R.A.); 2 Frontier Science Research Center, University of Miyazaki, 1-1 Gakuenkibanadai-Nishi, Miyazaki 889-2192, Japan; 3 Faculty of Animal Science, Universitas Gadjah Mada, Jl Fauna 3, Yogyakarta 55281, Indonesia; nafiatul.umami@ugm.ac.id; 4 Faculty of Agricultural Technology, King Mongkut’s Institute of Technology Ladkrabang, Bangkok 10520, Thailand; pattama.ni@kmitl.ac.th; 5 Faculty of Agro-Based Industry, Jeli Campus, Universiti Malaysia Kelantan, Jeli 17600, Kelantan, Malaysia; mijanur.r@umk.edu.my

**Keywords:** genetic transformation, genome editing, protoplast, somatic embryogenesis, warm-season grass

## Abstract

Warm-season grasses are C_4_ plants and have a high capacity for biomass productivity. These grasses are utilized in many agricultural production systems with their greatest value as feeds for livestock, bioethanol, and turf. However, many important warm-season perennial grasses multiply either by vegetative propagation or form their seeds by an asexual mode of reproduction called apomixis. Therefore, the improvement of these grasses by conventional breeding is difficult and is dependent on the availability of natural genetic variation and its manipulation through breeding and selection. Recent studies have indicated that plant tissue culture system through somatic embryogenesis complements and could further develop conventional breeding programs by micropropagation, somaclonal variation, somatic hybridization, genetic transformation, and genome editing. This review summarizes the tissue culture and somatic embryogenesis in warm-season grasses and focus on current status and above applications including the author’s progress.

## 1. Introduction

Forage grass plays a vital role in the successful operation for livestock production since ruminants are heavily dependent on forage for their feed and production [1]. Grasses are the main feed for ruminants, accounting for 60–90% of ruminant feed requirements worldwide [2]. By 2050, the human population is projected to reach 10 billion [3], and meat and milk consumption in developing countries is expected to at least double [4]. Therefore, there is a concern to increase forage productivity and quality for efficient livestock production. Grasses can be grouped into two large categories; warm- and cool- season grasses. Warm-season grasses are C_4_ plants and have a high capacity for biomass productivity. The grasses are cultivated mainly in the tropics, subtropics, and also in some warm temperate areas in the world. There are 4783 species of C_4_ plants in the grass family worldwide [5], and they are highly diverse, some of which are economically important crops: maize (*Zea mays*), sorghum (*Sorghum bicolor*), and sugarcane (*Saccharum officinarum*). Bahiagrass (*Paspalum notatum*), *Brachiaria* grass (*Urochloa* spp.), Guinea grass (*Panicum maximum*), Napier grass (*Pennisetum purpureum*), Rhodes grass (*Chloris gayana*), etc. are used for grazing, forage and silage, and Bermuda grass (*Cynodon dactylon*), *Paspalum* grass (*Paspalum* spp.), *Zoysia* grass (*Zoysia* spp.) are used as turf and greening plants. Maize and sugarcane are also used in bioethanol production. Recently, the high productivity of warm-season grasses has focused on the use of lignocellulosic biomass, and there have been studies on switchgrass (*Panicum virgatum*) and *Miscanthus* spp. as model bioenergy crops for sustainable energy production.

Many important warm-season perennial grasses multiply either by vegetative propagation or form their seeds by an asexual mode of reproduction called apomixis. The possibility of improving these plants by conventional breeding methods depends on the availability of natural genetic variation and its manipulation through breeding and selection. However, apomictic grasses have breeding barriers for hybridization and there are naturally not many genetic variations. Therefore, it is difficult to adapt to conventional breeding by crossbreeding in many warm-season grasses. Biotechnology involving plant tissue culture is a powerful complementary tool in conventional plant breeding programs [6]. Major categories of these methods can be summarized as induction and screening of desirable mutants at the cellular and tissue level, somatic hybridization between remotely related species, induction of haploid plants as breeding materials, and genetic transformation in protoplasts and plant tissues, as well as micropropagation of unique genotypes. However, in general, in vitro culture in warm-season grasses is not easy, and few grass species have established sufficient tissue culture systems [7].

There are two processes of plant regeneration, namely organogenesis and somatic embryogenesis. In general, organogenesis involves the sequential formation of shoots and roots from tissues, depending on the appropriate culture conditions. On the other hand, somatic embryogenesis is a developmental process in which plant somatic cells dedifferentiate to become totipotent embryonic stem cells with the ability to produce embryos. This new embryo can further develop into a whole plant [8]. Since the first description of somatic embryogenesis in the cell culture of carrots (*Daucus carota*) [9], this process has been reported in various plant species [10,11,12,13,14]. A unique characteristic of the somatic embryo is its continuous proliferation, as development is never arrested [15]. Therefore, somatic embryogenesis represents a powerful tool for mass production, germplasm conservation, protoplast culture and genetic improvement in plant species.

This review covers tissue culture and somatic embryogenesis including the influence of explants and culture condition. It also describes their application to breeding techniques such as protoplast culture, somaclonal variation, genetic transformation, and genome editing, including the authors’ progress.

## 2. Tissue Culture and Somatic Embryogenesis

To date, an effective in vitro regeneration system in cool-season grasses has been reported in many species using various explants and culture conditions. On the other hand, a few grass species have established sufficient tissue culture systems in warm-season grasses [16]. Despite a number of in vitro regeneration systems having been reported in recent years, most of these regeneration systems are based on somatic embryogenesis (Table 1). In warm-season grasses, callus induction and somatic embryogenesis are important points for establishing efficient tissue culture systems. In the Gramineae, the first successful attempt was made in barley (*Hordeum vulgare*) [17] where somatic embryos were formed on the scutellum of cultured immature zygotic embryos. Somatic embryogenesis in warm-season grass was first reported in Guinea grass [18] and pearl millet (*Pennisetum glaucum*) [19], followed by reports of somatic embryogenesis and plant regeneration in most of the important species.

There are two different modes of somatic embryogenesis: direct somatic embryogenesis and indirect somatic embryogenesis [88]. In warm-season grasses, indirect somatic embryogenesis is mostly observed, and somatic embryos are usually induced through callus formation. Therefore, the induction of callus forming somatic embryos (embryogenic callus) is the most important step to establish an efficient tissue culture system. At the stage of embryogenic callus formation, various factors affect its efficiency and quality, including the genotype of the donor plant, the explant type, the media, and plant growth regulators.

**Figure 1:**
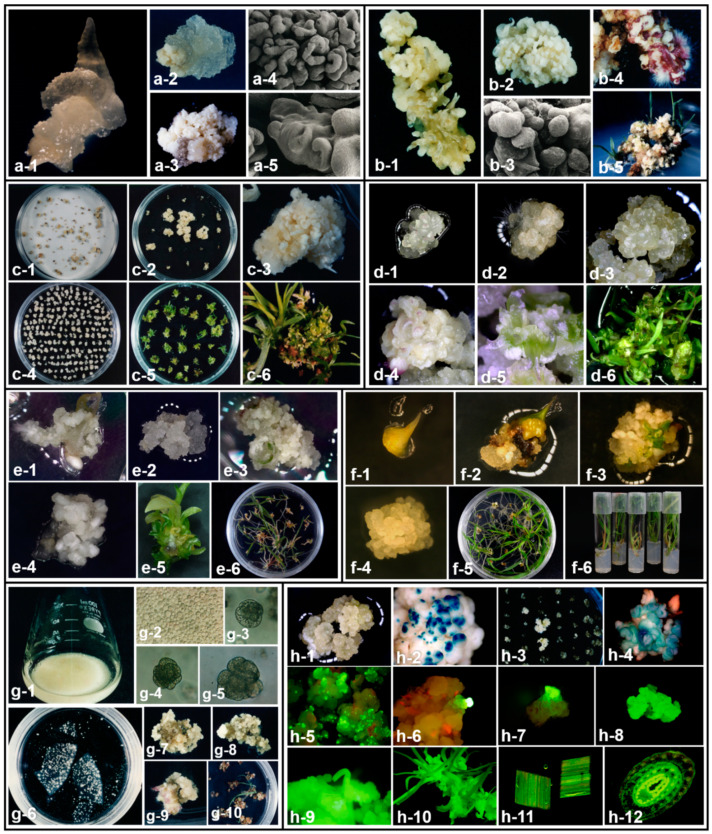
Somatic embryogenesis and its application to protoplast culture and genetic transformation in some warm-season grasses. (**a**) Somatic embryogenesis from immature zygotic embryos in Guinea grass (*Panicum maximum*). (1) Immature zygotic embryo, (2) non-embryogenic callus, (3) embryogenic callus, (4,5) SEM of somatic embryos at different stages of development. (**b**) Somatic embryogenesis from immature inflorescences and plant regeneration in dallisgrass (*Paspalum dilatatum*). (1) Primary callus after 14 d of culture, (2) embryogenic callus, (3) SEM of pro-embryogenic structures, (4,5) plant regeneration from somatic embryos. (**c**) Somatic embryogenesis from mature seeds and plant regeneration in bahiagrass (*Paspalum notatum*). (1) Primary callus after 14 d of culture, (2,3) embryogenic callus after 28 d of culture, (4) A sub-cultured micro-callus after 60 d culture, (5,6) Plant regeneration from micro-callus. (**d**) Developmental stages and plant regeneration of highly regenerative embryogenic callus cultured in CuSO_4_-supplemented medium. (1–3) Embryogenic callus cultured after 0 (1), 3 (2), and 14 (3) d on CuSO_4_ additional medium, (4,5) Shoot germination with scutellum formation, (6) elongation of germinated shoot. (**e**) Somatic embryogenesis from mature seeds and plant regeneration in ruzigrass (*Urochloa ruziziensis*). (1) Primary callus after 14 d of culture, (2–4) three types callus after 30 d of culture, non-embryogenic callus (2), friable embryogenic callus (3), compact embryogenic callus (4), (5). Plant regeneration from embryogenic callus, (6) rooting. (**f**) Somatic embryogenesis from apical meristem and plant regeneration in Napier grass (*Pennisetum purpureum*). (1) Apical meristem, (2,3) primary callus after 10 (2) and 45 (3) d of culture, (4) compact and proliferating uniform embryogenic callus. (5) Plant regeneration from embryogenic callus, (6) rooting. (**g**) Cell colony formation and plant regeneration from suspension protoplasts of dallisgrass. (1) Typical suspension cells, (2) isolated protoplasts from suspension cells, (3–5) cell division and cell colony formation from protoplasts after 5 (3), 7 (4) and 10 (5) d of culture, (6) colonies formed from protoplasts after 20 d of culture, (7,8) somatic embryos formation from protoplast-derived colonies, (9,10) plant regeneration from somatic embryos. (**h**) Stable transformation of bahiagrass mediated by particle inflow gun with bialaphos screening (1–4) and GFP (green fluorescent protein) visual screening (5–12). (1) Highly regenerative embryogenic callus for target tissue, (2) transient GUS (β-glucuronidase) expression 16 h after bombardment, (3) Bialaphos resistant callus under selection. (4) Stable GUS expression on bialaphos resistant callus. (5) Transient GFP expression 16 h after bombardment, (6,7) GFP expressing callus 14 d after bombardment, (8–10) GFP expression from transformed callus to plant regeneration, (11,12) GFP expression in leaf (11) and stem (12) of transgenic plants.

Cell totipotency is the most important characteristic of plant cell cultures, but not all cells are totipotent. For this reason, immature zygotic embryos and immature inflorescence that are capable of somatic embryogenesis with high potential for cell division are often used as explants in a wide range of cereal plants. Similar tissues are often used in warm-season grasses, and the authors have succeeded embryogenic callus induction and plant regeneration using immature zygotic embryos in Guinea grass [40] (Figure 1a) and immature inflorescences in dallisgrass (*Paspalum dilatatum*) [47] (Figure 1b). These tissues have high potential for embryogenic callus formation and provide high quality material in tissue culture. However, immature and developing tissues have seasonal limitations as materials. Monocotyledonous plants have limited explant sources that can form somatic embryos compared to dicotyledons. In addition to the above tissues, apical meristems, mature seeds (mature zygotic embryos), axillary buds of internodes, and germinated plants are mainly used, and hypocotyls and young leaves are used for callus induction in some cases (Table 1). Among them, mature seeds are the preferred alternatives to immature embryos in warm-season grasses since they can be stored for a long time and could be used any time without seasonal limitation. However, the frequency of embryogenic callus formation in warm-season grasses is low, mainly due to the fact that most seeds are of the outcrossing mode of reproduction and genetic heterogeneity is strong which consequently prevents the induction of uniform, high-quality embryogenic callus. Therefore, the authors have devised a two-step callus induction method in which a large number of seeds are sown on filter paper soaked with liquid medium to induce primary callus and then sub-cultured on solid medium to produce embryogenic callus (Figure 1c). By using this method, it is possible to select high quality embryogenic callus lines from a large number of seeds, and to provide materials with high regeneration capacity for genetic transformation [89].

Plant propagation and regeneration by in vitro culture is driven by the assimilation of ions such as nitrogen, phosphate, magnesium, and calcium. The Murashige and Skoog (MS) medium is now widely used for plant tissue culture in most warm-season grasses [90]. MS medium provides the basic nutrients, with the addition of maltose and sorbitol to regulate osmotic pressure, thiamine, L-glutamine, nicotinic acid, casein, proline as amino acids, and AgNO_3_ as ethylene inhibitor, and CuSO_4_ as useful microelements, which are effective in somatic embryogenesis [16]. In bahiagrass, CuSO_4_ is effective for somatic embryogenesis, and changing it to 50 μM from 0.1 μM in normal MS medium enhanced somatic embryogenesis frequency and produced high quality embryogenic callus, compact and dense with pro-embryos (Figure 1d). This modified culture minimized the problems associated with the loss of regeneration and increase in albinism which frequently occur in long term cultures of warm-season grasses [60,68,89].

Plant growth regulators are needed to control callus formation, proliferation, somatic embryo formation, plant regeneration, and rooting. Auxins [2,4-dichlorophenoxyacetic acid (2,4-D), dicamba, 1-naphtaleneacetic acid (NAA), indole-3-acetic acid (IAA), Indole-3-butyric acid (IBA), picloram] and cytokinins [6-benzylaminopurine (BAP), kinetin (KN), zeatin] were used for in vitro culture in a wide range of plant species. 2,4-D is used for callus induction, somatic embryogenesis, and proliferation in most warm-season grasses. It is often applied at 2–10 mg L^−1^, and is combined with low concentrations of cytokinins to control somatic embryogenesis. For plant regeneration, BAP is often used at concentrations of 1–3 mg L^−1^, and low concentrations of NAA added, or sometimes hormone-free media.

The authors have established tissue culture systems for several warm-season grasses, from callus induction to plant regeneration. Embryogenic callus was initiated from immature embryos on MS medium supplemented with 10 mg L^−1^ 2,4-D, 10% coconut water and solidified with 0.8% agar in Guinea grass (*Panicum maximum*) (Figure 1a). Initially various types of calli were obtained and embryogenic responses were found to be correlated with the genotypes investigated. For somatic embryo germination and plant formation, MS medium supplemented with gibberellic acid and kinetin were used. The twelve genotypes analyzed can be classified into three groups by the frequency of somatic embryo formation and degree of apomixis. One of the groups consisted of highly apomictic genotypes with a high embryogenic capacity [40]. Plant regeneration from cultured immature inflorescences of dallisgrass (*Paspalum dilatatum*) was obtained by somatic embryogenesis (Figure 1b). Embryogenic callus was initiated from immature inflorescences on MS medium supplemented with 2, 5, and 10 mg L^−1^ 2,4-D and solidified with 0.2% Gellan Gum. Somatic embryos developed and germinated precociously when embryogenic calli were transferred to a medium containing kinetin and gibberellic acid. All regenerants were successfully grown to maturity [47]. In bahiagrass, embryogenic callus was initiated from mature seeds on MS liquid and solid medium supplemented with 2 mg L^−1^ 2,4-D with a two-step callus induction method (Figure 1c). Selection of high-quality callus from a large number of mature seeds could be obtained by this modified culture. In addition, the selected good quality callus was cultured in MS medium with 2 mg L^−1^ 2,4-D, 0.1 mg L^−1^ BAP and 50 μM CuSO_4_, which resulted in dense pro-embryos on the surface of the callus and maintained high potential regeneration capacity for long-time culture (Figure 1d) [89].

*Urochloa* species are widely cultivated in tropical and subtropical regions, and are utilized as a main forage grass in South America. Ruzigrass (*Urochloa ruziziensis*) is one of several diploids with a sexual reproduction mode in the *Urochloa* genus, and we have established tissue culture system for this species. Embryogenic callus was induced from mature seeds on MS medium containing 4 mg L^−1^ 2,4-D and 0.2 mg L^−1^ BAP. Plant regeneration was achieved by culturing on MS medium with 2.0 mg L^−1^ BAP and 0.1 mg L^−1^ NAA (Figure 1e) [73]. However, in long-term tissue culture periods, spontaneous appearances of polyploids (tetraploid and octoploid) in plants regenerated from embryogenic calli were reported. At present, it is recommended that two-months-old or younger embryogenic calli are best suited for ruzigrass transformation since these calli generate fertile diploid plants [91]. As application for the breeding program, a tetraploid ruzigrass was produced by colchicine treatment of in vitro multiple-shoot clumps or in vitro germinated seedlings [92]. Subsequently, a new cultivar ‘Isan’ produced from the hybrids between the tetraploid ruzigrass and Mulato was selected for variety registration and investigation for initial growth (degree of plant growth between two to four weeks after sowing) and vigor in the tropical islands of Okinawa, Japan [93]. Likewise, the tetraploid ruzigrass have expanded the breeding material of the genus *Urochloa* by crossing it with tetraploid apomixis cultivars [94,95]. Napier grass (*Pennisetum purpureum* Schumach.) is a highly productive C_4_ tropical forage grass that has been targeted as a high potential bioenergy crop. Apical meristems were used as explants and cultured on MS medium with 2 mg L^−1^ 2,4-D, 0.5 mg L^−1^ BAP, and 50 μM CuSO_4_ in four accessions. A dwarf type with late-heading (DL line) had the best response for embryogenic callus formation. Highly regenerative calli that formed dense polyembryogenic clusters were selected through 14 d interval of subculture and maintained regeneration for six months (Figure 1f) [68]. These culture systems based on somatic embryogenesis are fundamental techniques for protoplast culture, genetic transformation and genome editing in warm-season grasses.

## 3. Somatic Embryogenesis Related Genes and Relationship to Apomixis in Warm-Season Grasses

Various genes that are involved in somatic embryogenesis include (a) housekeeping genes [96], (b) auxin-inducible genes, (c) ABA inducible genes [97], (d) transcription factors [98], (e) homeobox genes [99,100,101], and (f) maturation genes [102]. Apomixis, an asexual mode of reproduction of avoiding meiosis, is abundant in warm-season grasses and can produce seeds of the same genotype. Apomixis has the potential to maintain hybrid vigor for many generations in economically important plant genotypes. The evolution and genetics of asexual seed production are unclear, and much more effort will be required to determine the genetic architecture of this phenomenon. Somatic embryonic receptor-like kinases (SERKs) consist of plasma membrane receptor genes that have been characterized in a variety of species and have been found to be associated with several aspects of plant development, including reproduction. The expression of SERK is observed from competent cell stage up to the globular and heart stage of somatic embryos [103]. In Guinea grass, highly apomictic genotypes indicated a high embryogenic capacity [40], which suggested the involvement of a similar gene in somatic embryogenesis and apomictic seed formation. *SERK* genes are involved in another development and in competent cell stage up to the globular stage of somatic embryos and early embryo development in sexual and asexual seed formation in *Paspalum notatum* [104] and *Urochloa genus* [105]. Stronger expression of *PnSERK*2 in embryogenic calli of apomicts compared to those of sexual plants suggested an association with apomixis [104]. However, SERK3 was differentially expressed from other *SERKs* and was possibly down regulated in associated with apomictic development [105]. Somatic embryogenesis and apomixis embryo development have many similarities, and in addition to the *SERK* gene, *BBM*, *LEC1*, *LEC2*, *LEC3*, *FUS3*, etc. have common functions [106,107]. Warm-season grasses are suitable materials for studying apomixis, and it is essential to accumulate genetic information on somatic embryogenesis and use them as candidate genes for isolating apomixis-related genes.

## 4. Suspension Cell and Protoplast Culture

Suspension cell culture uses single cells or small aggregates of cells that multiply while suspended in agitated liquid medium. The establishment of single cell cultures in warm-season grasses has given excellent opportunity to develop somatic embryos and support protoplast systems. Moreover, suspension cell culture allows accelerated culture of embryogenic cells, including transgenic cells, which could lead to somatic embryos and their regeneration [108]. Such systems and protocols have been fundamental towards advancing breeding objectives especially in tropical grasses with economic importance to bioenergy, forage feed, cereals, and turfgrass. Suspension culture protocols have been established in many warm-season grasses including pearl millet [59], Guinea grass [109], dallisgrass [47], bahiagrass [13], zoysiagrass [87], Bermuda grass [110], and switchgrass [111] etc. With the current trends in “omics” and genome editing technologies, there have been a high preference to use single cells such as protoplast for DNA delivery and regeneration; and in that suspension culture is an indispensable tool to achieve such platforms [112,113,114,115].

Suspension cell-derived protoplasts that regenerate via somatic embryogenesis have been reported in few warm-season grasses such as switchgrass (*P. virgatum* L.) [111] and Guinea grass (*Panicum maximum*) [80]. However, some reported the failure of protoplast-derived microcalli to regenerate despite high protoplast yield from Finger millet (*Eleusine coracana*) [116]. Also, high exposure to enzyme treatment could cause cell toxicity and mitotic disorder in pearl millet (*P. glaucum*) [117]. Prior to protoplast isolation from dallisgrass, suspension cells previously derived from immature embryo-derived calli were conditioned with MS liquid medium without sucrose and growth regulators [83]. This treatment resulted to an increase in protoplast yield and colony formation. Embryogenic structures could be maintained proliferating in suspension culture only between one to two months to avoid the loss of regeneration. On the other hand, recalcitrance to plant regeneration has been said to be a major bottleneck in the application of protoplast in many warm-season grasses [111]. Factors such as genotype, source of explant, isolation method, culture medium, and the physical environment affect regeneration [118].

Protoplast fusion and somatic hybridization offers the potential to produce novel crops and overcome breeding obstacles in polyploid and apomictic warm-season grasses [7,119,120,121]. It could provide alternative ways to produce hybrids from sexually incompatible species and offers opportunity for intergeneric hybridization [16]. Although protoplast fusion was more successful in Solanaceae family, such approach has produced somatic hybrids in Gramineae species including wheat (*Triticum aestivum*) and maize (*Zea mays*) [122,123], pearl millet (*Pennisetum americanum*) and sugarcane (*Saccharum officinarum*) [124,125], Guinea grass (*Panicum maximum*) and dallisgrass (*Paspalum dilatatum*) [119], and Guinea grass (*Panicum maximum*) and pearl millet (*Pennisetum americanum*) [126]. On the other hand, C_4_ photosynthesis genes could be introduced in C_3_ crops by somatic hybridization as demonstrated by the protoplast fusion of C_4_ *Z. mays* and C_3_ *Triticum* sect. *trititrigia* MacKey [127] which formed somatic hybrids. Also, protoplast fusion between C_3_ rice and C_4_ Guinea grass produced somatic hybrids that exhibited abnormal floral structure and low fertility [128]. On the other hand, fusion of pearl millet and oat (*Avena sativa*) produced haploid embryos and karyoptically stable hybrids [129,130]. Although somatic hybridizations between C_3_ and C_4_ grasses have been moderately successful [131], these reports show the capacity for gene transfer between C_4_ warm-season and C_3_ cool-season grasses by protoplast fusion.

Gene-editing technology has great potential for efficient and accelerated improvement in warm-season grasses. Recently, there has been a renewed interest in using protoplasts in gene silencing and genome editing using CRISPR technologies [132,133,134,135]. Due to the naked nature of protoplasts, it makes it an ideal material for direct gene transfer to individual plant cells. Cereal crops and few forage grasses utilized protoplasts to evaluate CRISPR systems including maize [112], millet [112], sorghum [114], Zoysia [136], and switchgrass [132,137]. Transgene-free edited plants have used CRISPR–Cas RNP delivery using protoplasts [138], and recently, Banakar et al. [139] developed the protoplast-based RNP delivery approach and successfully demonstrated it in dicots and monocots, including *Setaria viridis*. The use of protoplast with new plant breeding technologies could offer more precise results and unique advantages for bioenergy and forage crops [118,140].

## 5. Somaclonal Variation

The application and advancements in somatic embryogenesis in tissue culture has made regeneration possible for various recalcitrant warm-season grass species in vitro for mass production, embryo rescue, and breeding. Culture and preservation of elite genotypes, which are selected for their superior traits, need a high degree of genetic uniformity amongst the regenerated plants. However, cells and tissues that have been subjected to long-term culture and repetitive stresses may lead to the production of somaclonal variation (SV), a genetic variability caused by gene mutation or changes in epigenetic marks. SV is a genetically stable variation produced in plant tissue culture and has been useful in creating novel variants [16,141,142], and as strategy in overcoming strict transgenic regulations [143]. It has also been an alternative tool to increase genetic variation specially when there is a narrow genetic base such as apomictic species.

Propagation, breeding and genetic improvement in warm-season grasses require the selection of important genotypes from diverse genetic resources. With an effort to isolate somaclonal variants, Li et al. [144] reported a large-scale tissue culture that regenerated approximately 7900 St. Augustine grass ‘Raleigh’ (*Stenotaphrum secundatum*) plants in vitro and characterized 119 morphological variants which focused on plant variants that had semi-dwarf growth habit and still maintained growth vigour. Somaclonal variants with improved agronomic traits include high seed sets in *Paspalum dilatatum* [145], fall-army worm resistance in *Cynodon dactylon* ‘Brazos R3′ [146,147], herbicide resistance in seashore paspalum (*Paspalum vaginatum*) [143,148] and freezing tolerance in St. Augustine grass (*S. secundatum*) [149], seashore paspalum (*P. vaginatum*) [150], and centipedegrass (*Eremochloa ophiuroides*) [151,152]. Likewise, a somaclonal triploid Bermuda grass (*Cynodon transvaalensis × C. dactylon*) with increased drought tolerance was obtained following 2-year cell suspension culture and subsequent regeneration of somatic embryos [110,153]. Grasses have tremendous potential for phytoremediation of trace element-polluted soils [154], and breeding by in vitro culture can be a feasible approach to enhancing heavy metal accumulation properties such as lead uptake as reported in *Cynodon dactylon* [155]. Considered an important breeding approach that could increase genetic diversity and expand germplasm pool, there is yet more to explore on somaclonal variation for the development of new and improved cultivars in warm season grasses.

## 6. Genetic Transformation

To develop molecular breeding of plants, the establishment of tissue culture systems, genetic transformation technology, and the isolation of useful genes are three critical points. In the case of warm-season grasses, only few species have sufficiently established these systems. One of the main reasons for this is the need to establish a stable and efficient tissue culture system and the choice of target tissues with competence for transformation. Target tissues for genetic transformation in warm-season grasses used embryogenic callus, suspension cell, protoplast, and stolon nodes [16]. Among them, embryogenic calli are frequently used for transformation, and their transformation efficiency greatly depends on their characteristics. Gondo et al. [89] found that callus culture with 50 μM CuSO_4_ resulted in the formation of compact callus with dense polyembryogenic clusters on the surface, and the modified callus shape produced a 3-fold increase in transient GUS expression. In addition, transformed callus could be recovered frequently and transgenic plants have been produced stably without loss of regenerative ability and increase in albinism.

Transgenic warm-season grass by direct gene transfer to protoplasts was first obtained in *Zoysia japonicus* [156]. Although transformation by protoplasts has been reported in temperate grasses; creeping bentgrass *(Agrostis stolonifera*), orchardgrass (*Dactylis glomerata*), tall fescue (*Festuca arundinacea*), Italian ryegrass (*Lolium multiflorum*) etc. [157], it is limited to a few warm-season grass species due to the difficulty in culturing protoplasts. As an alternative technology, microprojectile bombardment and *Agrobacterium*-based transformation have been developed and became the major methods for producing transgenic warm-season grasses (Table 2). Microprojectile particle gun is an effective transformation method for warm-season grass since it has let to introduce foreign genes into any cell or tissues without protoplast culture and *Agrobacterium* host specificity. *Agrobacterium*-mediated method is also applicable to several species due to the improvement of vectors [158,159,160] and has become the major genetic transformation method for some species due to its stability of gene insertion, low cost and simplicity (Table 2).

In the last two decades, genetic transformation has been successfully carried out in many species and produced several transgenic plants with agronomically useful genes in warm-season grasses (Table 2). The target genes are also highly dependent on the utilization of the grass species, which in the case of warm-season grasses is characterized by a wide range of uses, including forage grass, turfgrass, and bioenergy. Many warm-season grasses have high biomass but have low forage quality, and requires a continuous selection and breeding for improved forage quality. In bahiagrass, transgenic plants carrying *CAD* (cinnamyl alcohol dehydrogenase) gene for lignin biosynthesis with a RNAi vector were developed. The CAD down-regulated transgenic lines had significantly reduced lignin content and improved digestibility within 5–10% [196]. Transgenic plants overexpressing the wheat-derived fructan synthesis genes, sucrose: sucrose 1-fructosyltransferase (1-SST) gene and sucrose: fructan 6- fructosyltransferase (6-SFT) gene, have also been produced. Fructan biosynthesis, a metabolic property found in cool-season grasses, was established for the first time in warm-season bahiagrass as transgenic plants generated fructan and consequentially increased sugar content [194].

Bioenergy refers to renewable energy from biological sources. Lignocellulosic ethanol, a second-generation biofuel, has the potential to fill most global transportation fuel needs and does not present a conflict between energy demands and the food supply [215]. More importantly, grass biomass is one of the world’s most productive and sustainable lignocellulosic bioenergy sources [216,217]. Decreasing lignin content and increasing sugar content will lead to efficient bioethanol production, which is the same breeding strategy for improving forage grass quality. An efficient transformation system has been established in switchgrass (*Panicum virgatum*), a model bioenergy crop, and is reported to down-regulate many lignin synthesis genes and its transcription factors genes [218]. Transformation systems in other grass species with higher biomass production, such as *Miscanthus sinensis* [169,170] and Napier grass (*Pennisetum purpureum*) [68], have also been developed. Warm-season grasses with fine texture and dense growth are used as turfgrasses in parks, gardens, golf courses, and sports ground. They are also industrially important and are used as greening materials in various situations. In particular, *Zoysia*, *Cynodon*, and *Paspalum* are the most common turfgrasses, and transgenic plants have been successfully produced (Table 2). Environmental stress resistance is an important trait for turfgrasses, and drought-resistant transgenic plants over-expressing *DREB* and *WRKY* transcription factors and *GA2ox1* gibberellin synthesis gene for drought resistance, and those with *ICE1* and *CBF1* transcription factors for cold tolerance were developed (Table 2).

The authors have established genetic transformation systems through somatic embryogenic cultures in the following warm-season grasses. Bahiagrass (*Paspalum notatum*) is a typical warm-season pasture grass, this transformation system employs a marker selection with *bar* genes [89] and a visual screening with *GFP* gene [192] (Figure 1h). Both methods produce stable transgenic plants with around 3.0% transformation efficiency, which is similar to other apomictic warm-season grasses [7]. Other transformation systems have been established in ruzigrass [91], which is widely cultivated in South America, and Napier grass [68] which is utilized as forage and bioenergy crops. In Rhodes grass, which is used for hay production and silage, transgenic plants have been successfully produced through organogenesis by using multiple shoot clumps as the target tissue [162]. Thus, we have successfully developed genetic transformation systems for many warm-season grasses by applying the appropriate culture system for each species.

## 7. Genome Editing

The genome editing is a breakthrough technology that can cut a targeted sequence at a pinpoint with artificial nucleases (ZFN, TALEN etc.) and RNA-inducible nucleases (CRISPR/Cas9), and can perform gene knockout and knock-in of target genes. The technology is being used in various research fields such as medicine, industry, science, and agronomics. In the field of plants, it is focused as a new breeding technology different from genetic transformation. On the other hand, the way of regulation of genome editing is discussed in the world.

Recently, highly functional soybeans with high oleic acid, developed by genome editing, have actually been produced without under the GM regulation and sold commercialization as soybean oil in the US [219]. Most of countries, except the EU and New Zealand, have accepted the technology and adapted legislations to these technologies or released guidelines supporting the use of genome editing [140]. This technology of plants is applied not only to model plants but also to crops such as corn, wheat, and sorghum [220,221]. Although the research has been advanced for practical use, genome editing of forage and turf grass has been successful in a few species such as *Lolium perenne* [185] and switchgrass [137,222].

In common, plant genome editing technology was performed to insert the CRISPR/Cas9 vector into the genome. The genome-edited mutant has the inserted vector in its genome, so the vector must be removed at the next generation by self-pollination. Therefore, this genome editing system is difficult to apply to forage grass or turfgrass due to their varying reproductive modes which include vegetative propagation, apomixis, and cross fertilization. In recent years, new genome editing systems have been developed which introduce Cas9 protein-gRNA ribonucleoproteins (RNPs) into plant cells and make genome editing [223]. This technique is completely free of DNA so the risk of transgene integration into the genome can be excluded. It is expected that this technology will be applied to many plant species. Although this system has been used mainly with protoplasts in plants, it has not been possible to apply it to a wide range of plant species due to the difficulty for plant regeneration. On the other hand, genome editing using immature zygotic embryos has been successfully achieved by introducing RNPs with a particle bombardment in wheat [224,225,226], and maize [227,228], but this method cannot be applied to some warm-season grasses with a vegetative propagation and a hard to produce seeds.

The authors are working on a new genome editing system for warm-season grass and turf, focusing on somatic embryos as an alternative target tissue, which have high transformation efficiency and vigorous cell division with high potential of regeneration. In bahiagrass somatic embryo culture system, high dense pro-embryos on the surface of the callus can be continuously renewed by forming and proliferating secondary somatic embryos. Similar to genome editing of immature zygotic embryos, RNPs are introduced into somatic embryo cells by particle bombardment. Immediately after the RNPs are introduced into the cells, the genome-edited events occur at the cellular level, but through the culture system above, the cells can be developed into somatic embryos and regenerated into new plants. In our current research, we have already confirmed mutation at the target site at the cellular level after the delivery of RNPs into the somatic embryos of bahiagrass (unpublished). This genome editing method is completely free of DNA introduction and can be applied to vegetative propagated plants and apomictic plants, especially included warm-season grasses, and could be a novel method to produce genome-edited plants in its generation.

## 8. Conclusions

In the recent two decades, tissue culture systems have been established in many warm-season grasses. Also, there have been many reports of genetic transformation, which hardly succeeded before. Most of the plant regenerations are based on somatic embryogenesis, and a stable and efficient culture system ensures the applications to micropropagation, protoplast culture, genetic transformation and genome editing. In recent years, whole genome sequences have been determined in foxtail millet (*Setaria italica*) [229], switchgrass [230], *Miscanthus sinensis* [231], and *Zoysia* spp. [232], and progress in research using genomic information is expected in warm-season grasses, where genetic information has been limited until now. In particular, the genes for somatic embryogenesis are deeply involved in apomixis seed formation, thus exploration and identification of these candidate genes are expected to be applied to future breeding technology. In addition, genome editing technology is being applied to warm-season grasses, and the practical genomic breeding, unlike genetic transformation, is becoming possible. Our research team has been developing molecular breeding of some warm-season grasses, and has established a step-by-step process starting from somatic embryo formation, plant regeneration, suspension culture, protoplast culture, and genetic transformation. At this stage, we have just begun to focus on practical and applied research. We are now working on genome editing using genetic information and will develop new breeding materials in the next stage.

## Figures and Tables

**Table 1:** Summary of tissue and protoplast cultures in warm-season grasses.

Plant Species	Explants Source ^1^	Plant Regeneration ^2^	References
**Callus Induction and Plant Regeneration**			
*Bouteloua gracilis*	AM	SE	[20]
*Cenchrus ciliaris*	II	SE	[21]
	MS, AM, II	SE	[22]
*Chloris gayana*	SL	OR, SE	[23]
*Cynodon dactylon*	II	SE	[1,24,25,26]
*Digitaria sanguinalis*	AM	SE	[27]
*Eragrostis tef*	SL	SE	[28]
	MS	SE	[29]
*Imperata cylindrica*	AM, II	OR	[30]
	AM	OR	[31]
*Miscanthus sinensis*	NS	OR	[32]
	AM	OR, SE	[33]
*Panicum* spp.	AM, II, MS	OR	[34]
	MS	SE	[35,36]
*Panicum bisulcatum*	MS	OR, SE	[37]
*Panicum maximum*	L	SE	[18,38]
	II, IE, ME	OR, SE	[39]
	IE	SE	[40]
*Panicum miliaceum*	M	OR	[41]
	II	SE	[42]
*Panicum sumatrense*	II	SE	[42]
*Panicum virgatum*	II, L	SE	[43]
	II	SE	[44,45]
*Paspalum* spp.	II	OR	[46]
*Paspalum dilatatum*	II	SE	[47]
*Paspalum notatum*	IE, ME	SE	[48]
	MS	SE	[13,49,50]
	SL	SE	[51]
*Paspalum scrobiculatum*	M	OR	[52]
	IE	SE	[53]
	IE, ME	SE	[54]
	MS	OR	[55]
	SL	SE	[56]
*Paspalum vaginatum*	II	SE	[57]
*Pennisetum americanum*	II, IE	SE	[19,58]
	II	SE	[59]
*Pennisetum americanum* × *Pennisetum purpureum*	II	SE	[19]
*Pennisetum glaucum*	AM	SE	[60]
	SL	OR	[61]
	IE	SE	[62]
	AM, II, MS	OR, SE	[63]
*Pennisetum purpureum*	L	SE	[64]
	II	SE	[65,66]
	AM	OR	[67]
	AM	SE	[68]
*Setaria italica*	II	SE	[69]
	MS	OR	[70]
*Urochloa brizantha*	NS, MS	OR, SE	[71]
	MS	OR, SE	[72]
*Urochloa ruziziensis*	SL	OR, SE	[73]
*Zoysia japonica*	MS	SE	[74,75,76]
*Zoysia matrella*	II, NS	SE	[77]
	NS	SR	[78]
**Protoplast culture ^3^**			
*Panicum maximum*		PL	[79]
		CL	[80]
*Panicum miliaceum*		PL	[81]
*Paspalum scrobiculatum*		PL	[82]
*Paspalum dilatatum*		PL	[83]
*Pennisetum americanum*		CL	[84]
		PL	[85]
*Pennisetum purpureum*		PL	[86]
*Zoysia japonica*		CL	[74]
		PL	[87]

^1^ AM, apical meristem; IE, immature embryo; II, immature inflorescence; L, leaf; M, mesocotyl; MS, mature seed; NS, nodal segment; SL, seedling. ^2^ OR, organogenesis; SE, somatic embryogenesis. ^3^ CL, callus; PL, plantlet.

**Table 2:** Summary of genetic transformation in warm-season grasses.

Plant Species	Transformation Method ^1^	Transgenes ^2^	Outcome ^3^	References
*Bouteloua gracilis*	PB	*npt*, *gusA*	PL	[161]
*Chloris gayana*	PB	*bar*, *gusA*	PL	[162]
*Cynodon dactylon*	PB AG	*hph*, *gusA* *hph*	PL PL	[163] [164]
*Cynodon dactylon* × *C. transvaalensis*	PB	*hph*	PL	[165]
*Digitaria sanguinalis*	PB	*bar*, *gusA*	PL	[166]
*Eragrostis tef*	AG	*npt*, *gusA*, *PcGA2ox*	PL	[167]
*Miscanthus sinensis*	PB AG AG AG	*hph*, *gfp* *bar*, *gfp* *npt*, *MsCOMT* *npt*, *gusA*	PL PL PL PL	[168] [169] [170] [171]
*Panicum meyerianum*	AG	*hpt*, *gusA*, *ddsA*	PL	[172]
*Panicum virgatum*	PB AG AG AG AG AG AG AG AG AG AG AG AG AG	*bar*, *gfp* *hph*, *PvCOMT* *hph*, *PvCAD* *bar*, *hph*, *gusA* *hph*, *Pv4CL* *hph*, *gusA*, *pporRFP* *hph*, *gfp* *hph*, *PvMYB4* *hph*, *PvBMY1*, *PvBMY3* *hph*, *gusA*, *PuP5CS* *hph*, *OsAT10* *hph*, *LpP5CS* *hph*, *pporRFP* *hph*, *vPIP2;9*	PL PL PL PL PL PL PL PL PL PL PL PL PL PL	[173] [174] [175] [176] [177] [158] [178] [179] [180] [181] [182] [183] [108] [184]
*Paspalum dilatatum*	PB PB	*bar* *npt*, *PdCCR*	CL PL	[185] [186]
*Paspalum notatum*	PB PB PB PB PB PB PB PB PB PB PB	*bar*, *bar*, *gusA* *npt*, *AtGA2ox1* *npt*, *AtHB16* *npt*, *HsDREB1A* *bar*, *npt* *gfp* *npt*, *HvWRKY38* *bar*, *1-SST*, *6-SFT* *bar*, *gfp* *bar*, *CAD*	PL PL PL PL PL PL PL PL PL PL PL	[187] [89] [188] [189] [190] [191] [192] [193] [194] [195] [196]
*Paspalum vaginatum*	AG AG	*hph*, *gusA* *hph*, *CdtNF-YC1*	PL PL	[197] [198]
*Pennisetum glaucum*	PB PB PB PB PB AG AG	*hph*, *gusA* *bar*, *gusA* *bar*, *gusA*, *egfp* *pat*, *afp* *bar*, *gusA*, *pin* *hph*, *gusA* *bar*, *mag*	CL PL PL PL PL PL PL	[199] [200] [201] [202] [203] [204] [205]
*Pennisetum purpureum*	PB	*bar*, *gusA*	PL	[68]
*Setaria italica*	AG AG	*hph*, *gfp* *hpt*, *ntp*, *gusA*	PL PL	[206] [207]
*Setaria viridis*	AG	*hph*, *pporRFP*	PL	[208]
*Urochloa ruziziensis*	PB	*bar*, *gusA*	PL	[91]
*Zoysia japonica*	PP AG AG AG AG	*hph*, *gusA* *hph*, *gusA* *cryIA(b)*, *hph*, *gusA* *bar*, *ICE1* *bar*, *gusA*, *AHLs*	PL PL PL PL PL	[156] [209] [210] [211] [212]
*Zoysia tenuifolia*	AG	*hph*, *gusA*	PL	[213]
*Zoysia sinica*	AG	*bar*, *CBF1*	PL	[214]

^1^ PB, particle bombardment; AG, *Agrobacterium*-mediated; PP, protoplast transformation. ^2^ *afp*, the antifungal protein from *Aspergillus giganteus*; AHLs, AT-hook motif nuclear-localized genes from *Arabidopsis thaliana*; *AtGA2ox1*, gibberellin 2-β-dioxygenase gene from *Arabidopsis thaliana*; *AtHB16*, homeobox gene from *Arabidopsis thaliana*; *bar*, phosphinothricin N-acetyltransferase gene from *Streptomyces hygroscopicus*; *CBF1*, a cold inducible transcription factor from *Arabidopsis thaliana*; *CdtNF-YC1*; a nuclear factor Y transcription factor from hybrid Bermuda grass (*Cynodon dactylon* × *Cynodon transvaalensis*); *CryIA(b)*, synthetic insecticidal protein genes from *Bacillus thuringiensis*; *ddsA*, decaprenyl diphosphate synthase gene from *Gluconobacter suboxydans*; *gfp*, green fluorescent protein gene form *Aequorea victoria*; *gusA*, β-glucuronidase gene from *Escherichia coli*; *hph*, hygromycin phosphotransferase gene from *Escherichia coli*; *HsDREB1A*, The dehydration-responsive element binding proteins gene from *Hordeum spontaneum*; *HvWRKY38*, WRKY transcription factor from *Hordeum vulgare*; *ICE1*, a regulator of cold-induced transcriptome from *Arabidopsis thaliana*; *LpP5CS*, proline biosynthesis gene from *Lolium perenne*; *npt*, neomycin phosphotransferase II gene from *Escherichia coli*; *mag*, a synthetic magainin gene from *Xenopus laevis*; *MsCOMT*, caffeic acid *O*-methyltransferase gene from *Miscanthus sinensis*; *OsAT10*, BAHD acyltransferase gene from *Oryza sativa*; *pat*, phosphinothricin N-acetyltransferase from *Streptomyces viridochromogenes*; *PcGA2ox*, GA inactivating gene from *Phaseolus coccineus*; *pin*, a synthetic prawn antifungal protein gene; *PdCCR*, cinnamoyl-CoA reductase gene from *Paspalum dilatatum*; *pporRFP*, red flourescence protein gene from *Porites porites*; *PuP5CS*, proline biosynthesis gene from *Puccinellia chinampoensis*; *PvCAD*, cinnamyl alcohol dehydrogenase gene from *Panicum virgatum*; *PvCOMT*, caffeic acid *O*-methyltransferase gene from *Panicum virgatum*; *PvMYB1*, *3*, *4*, transcriptional repressors of monolignol biosynthetic genes from *Panicum virgatum*, *PvPIP2;9*, aquaporin gene from *Panicum virgatum*; *Pv4CL*, 4-coumarate:CoA ligases gene from *Panicum virgatum*; *1-SST*, sucrose:sucrose 1-fructosyltransferase from *Triticum aestivum*; *6-SFT*, sucrose:fructan 6-fructosyltransferase from *Triticum aestivum*. ^3^ PL, Plantlet; CL, Callus.

## Data Availability

Not applicable.
